# Estimation of allele frequency and association mapping using next-generation sequencing data

**DOI:** 10.1186/1471-2105-12-231

**Published:** 2011-06-11

**Authors:** Su Yeon Kim, Kirk E Lohmueller, Anders Albrechtsen, Yingrui Li, Thorfinn Korneliussen, Geng Tian, Niels Grarup, Tao Jiang, Gitte Andersen, Daniel Witte, Torben Jorgensen, Torben Hansen, Oluf Pedersen, Jun Wang, Rasmus Nielsen

**Affiliations:** 1 Departments of Integrative Biology and Statistics, UC Berkeley, Berkeley CA 94720, USA; 2 Bioinformatics Centre, University of Copenhagen, Copenhagen, Denmark; 3 Beijing Genomics Institute, Shenzhen 518083, China; 4 Department of Biology, University of Copenhagen, Copenhagen, Denmark; 5 Beijing Institute of Genomics, Chinese Academy of Science, Beijing 101300, China; 6 The Graduate University of Chinese Academy of Sciences, Beijing 100062, China; 7 Novo Nordisk Foundation Center for Basic Metabolic Research, Faculty of Health Sciences, University of Copenhagen, Copenhagen, Denmark; 8 Hagedorn Research Institute, Copenhagen, Denmark; 9 Steno Diabetes Center, Gentofte, Denmark; 10 Faculty of Health Sciences, University of Copenhagen, Copenhagen, Denmark; 11 Research Centre for Prevention and Health, Glostrup University Hospital, Glostrup, Denmark; 12 Faculty of Health Sciences, University of Southern Denmark, Odense, Denmark; 13 Faculty of Health Sciences, University of Aarhus, Aarhus, Denmark; 14 Institute of Biomedical Sciences, University of Copenhagen, Copenhagen, Denmark

## Abstract

**Background:**

Estimation of allele frequency is of fundamental importance in population genetic analyses and in association mapping. In most studies using next-generation sequencing, a cost effective approach is to use medium or low-coverage data (e.g., < 15*X*). However, SNP calling and allele frequency estimation in such studies is associated with substantial statistical uncertainty because of varying coverage and high error rates.

**Results:**

We evaluate a new maximum likelihood method for estimating allele frequencies in low and medium coverage next-generation sequencing data. The method is based on integrating over uncertainty in the data for each individual rather than first calling genotypes. This method can be applied to directly test for associations in case/control studies. We use simulations to compare the likelihood method to methods based on genotype calling, and show that the likelihood method outperforms the genotype calling methods in terms of: (1) accuracy of allele frequency estimation, (2) accuracy of the estimation of the distribution of allele frequencies across neutrally evolving sites, and (3) statistical power in association mapping studies. Using real re-sequencing data from 200 individuals obtained from an exon-capture experiment, we show that the patterns observed in the simulations are also found in real data.

**Conclusions:**

Overall, our results suggest that association mapping and estimation of allele frequencies should not be based on genotype calling in low to medium coverage data. Furthermore, if genotype calling methods are used, it is usually better not to filter genotypes based on the call confidence score.

## Background

The frequency of an allele in the population is a fundamental quantity in human statistical genetics. This quantity forms the basis of many population and medical genetic studies. Many evolutionary forces change allele frequencies. Consequently, allele frequencies can be used to infer past evolutionary events. For example, allele frequencies at single nucleotide polymorphisms (SNPs) can be used to infer the demographic history of a population [1,2]. Patterns of allele frequency are also informative about the possible effects of natural selection. After a completed selective sweep, an excess of low-frequency and high-frequency derived SNPs is expected around the selected site [3-6]. Conversely, SNPs under the direct influence of negative selection are expected to be at lower frequency than predicted by demography alone [7,8]. Many commonly used summary statistics in population genetics like Tajima's *D *[9], Fu and Li's *D *[10], Fay and Wu's *H *[4] and *F_ST _* [11] are direct functions of allele frequencies. Allele frequencies also form the basis of association studies between SNPs and common disease. In their simplest form, case-control association studies seek to quantify the difference in allele frequency between cases (individuals with the disease) and controls (individuals without the disease) [12-14]. In particular, there has been rapidly growing interest in performing association studies between rare variants and common disease using data obtained from next-generation sequencing approaches [15-17].

Given the importance of allele frequencies in genetic studies, it is critically important to be able to estimate them reliably. Traditionally, allele frequencies were simply estimated by counting the number of times each allele had been seen in a sample from the population. This approach was often successfully used on SNP genotype data and Sanger sequencing data because the genotypes for each individual could often be unambiguously determined. However, this approach may fail when applied to data from next-generation sequencing technology. First, next-generation sequencing data has a higher error rate than traditional Sanger sequencing or SNP genotyping assays [18-20]. Second, in order to sequence more samples, researchers often sequence each individual at shallow coverage (e.g., [21]). Thus, each base will only be covered by a few reads, making it more difficult to accurately infer an individual's genotype at a particular site [20]. Finally, because the reads from next-generation sequencing technologies are often quite short, additional errors can occur when trying to align the short reads back to the reference genome [22,23]. For these reasons, estimating allele frequencies remains challenging.

Several different approaches have been proposed to attempt to make accurate inferences of allele frequency from next-generation sequencing technologies [24-31]. It is important to appreciate that no single approach has been consistently favored or endorsed by the community. Instead, a variety of approaches have been proposed and used by different scientific groups. The first set of approaches uses the traditional paradigm of estimating allele frequencies by first inferring individual genotypes [23,32-34] and then tabulating frequencies. Here, strict filters are used to attempt to account for the increased error rate and uncertainty inherent in the data [20,35]. Others have added linkage disequilibrium (LD) information and data from reference haplotypes to make more accurate genotype calls [21,36]. The second set of approaches seeks to directly estimate allele frequencies from the next-generation sequencing data without first attempting to infer genotypes [29-31]. These approaches have the advantage that they directly estimate the quantity of interest without first inferring other uncertain information (e.g., the individual genotypes). The utility of this type of approach has yet to be fully explored for different types of population and medical genetic studies.

Here we discuss the properties of a new likelihood approach designed to estimate the population minor allele frequency from next-generation sequencing data. We show that the new likelihood method can obtain accurate estimates of allele frequencies, even when the depth of coverage is quite shallow. Further, we show that the new likelihood method either performs as well as, or better than, genotype calling methods. Finally, we discuss the performance of the likelihood approach in testing for differences in allele frequency between cases and controls.

## Results

The minor allele is the less frequent allele in the population at a variable site. We first describe two main approaches to estimate the minor allele frequency (MAF) at a particular site in the genome. The first approach involves inferring individual genotypes and treating those inferred genotypes as being completely accurate when estimating the MAF. We then examine the performance of a likelihood framework that directly takes the uncertainty in assigning genotypes into account. Throughout our work, we assume that all segregating sites are biallelic.

### Estimation of MAF from called genotypes

One way to estimate the MAF from next-generation sequencing data is to first call a genotype for each individual using sequencing data, and then use those genotypes as if they are the true ones. This was the approach traditionally used for genotype data and Sanger sequencing data. It is not clear how well it will perform when applied to next-generation sequencing data.

A maximum likelihood approach can be used to infer the genotype for each individual from the next-generation sequencing data. At each site *j*, for each individual *i*, the likelihood for each of the three possible genotypes (assuming that we know the minor allele) is given as:(1)

where *D_i,j _* is the observed sequencing data in individual *i *at site *j*, *g_i_*_,_*_j _* ∈ {0, 1, 2} is the number of minor alleles contained in the genotype of each individual, and  and  control for sequencing errors and read base qualities, respectively. The observed sequencing data for each individual can be thought of as the alignment of reads at site *j *taking the read quality scores into account. This is represented as the genotype likelihood and is found in the genotype likelihood file (GLF) which is produced in many programs that analyze next-generation sequencing data, such as SOAPsnp and MAQ [23,32].

To assign a genotype to a particular individual, the likelihood of each of the three possible genotypes can be calculated for the individual. The genotype with the highest likelihood can then be assigned. However, researchers often prefer a more stringent calling criterion and will not assign a genotype to an individual unless the most likely genotype is substantially more likely than the second most likely one. Here the three possible genotypes are sorted by their likelihoods: , where *g*_(*k*) _corresponds to the genotype with the *k*th largest likelihood. With a given threshold *f*, one can call the genotype g_(1) _if . Otherwise, a genotype is not called and the individual's genotype is considered missing. A common threshold value of *f *is 1, indicating that the most likely genotype is at least 10 times more likely than the second most likely one. Note that this type of filtering may result in higher confidence for the "called" genotype, but it also results in more missing data.

### Maximum likelihood estimator of allele frequency

Instead of estimating the MAF from the called genotypes, a maximum likelihood (ML) method introduced by Kim et al. [30] (see also Lynch [29] for a similar approach) directly estimates MAFs and takes genotype uncertainty into account. Specifically, given a minor allele, the probability of observing the sequence data at each individual *i *is obtained by summing over the probabilities corresponding to all three possible genotypes.

Suppose that the three genotype likelihoods defined in Equation 1 are available. Using the same notation as above, let *D_j _* and *p_j _* be the observed sequencing data at site *j *and the corresponding MAF, respectively. The genotype probability given that minor allele frequency can be computed by assuming Hardy-Weinberg equilibrium (HWE). Then, assuming independence among individuals, the likelihood of the MAF at this locus is a product of all the likelihoods computed across all *N *individuals:(2)

The ML estimate of *p_j _* can be computed either by directly maximizing the likelihood for a restricted parameter space using the Broyden-Fletcher-Goldfarb-Shanno (BFGS) method [37-40] or by using the expectation-maximization (EM) algorithm [31,41]. When using the EM algorithm, the posterior expectation of a genotype is computed for each individual, and the mean of those posteriors is repeatedly updated. Our implementation of BFGS was faster than the EM algorithm. For example, to obtain estimates from 100,000 sites, BFGS took ~16 seconds but EM took ~100 seconds. However, the difference in speed may be implementation specific. In our case, for both methods, we stopped updating parameters when the increase in the likelihood was less than 0.001.

### Maximum likelihood estimator with uncertain minor allele

In practice, often the second most common nucleotide across individuals can be used as the minor allele. However, for rare SNPs (e.g., MAF < 1%), it is hard to determine which allele is the minor allele, since all four nucleotides may appear in some reads due to sequencing errors. To deal with this situation, we now describe a likelihood framework that takes the uncertainty in the determination of the minor allele into account.

Suppose that for site *j *we know the major allele *M*. Note that deciding which of two common alleles is likely to be the major one is not important since we are mostly concerned with estimating the frequencies at rare SNPs. Further, for alleles with intermediate frequencies (around 50%), the distinction between major and minor allele is less important. Assign the other three non-major nucleotides *m*_1_, *m*_2_, and *m*_3_. The likelihood introduced in Equation 2 assumes a fixed major allele *M *and fixed minor allele *m*. Therefore, to allow for uncertainty in the designation of the minor allele, the likelihood function can be modified as:(3)

Further, assuming that any of the three possible minor alleles is equally likely, we obtain:(4)

where . Since  can be very small with big data sets (e.g., with many individuals), it is useful to compute the likelihood in the log-scale. Order the three conditional log-likelihoods as to (*l*_(1)_*, l*_(2)_*, l*_(3)_), where *l*_(1) _is the largest one. Then,

### *G*-test using called genotypes for association mapping

In association studies, SNPs showing significant differences in allele frequency between cases and controls are said to be associated with the phenotype of interest. Association mapping can be performed using data from next-generation sequencing studies. We first discuss approaches that require calling individual genotypes and then perform a test for association using the called genotypes. In this approach, a genotype is first called for each individual. The genotypes can be filtered or unfiltered. Assuming independence across individuals and HWE, a 2 × 2 contingency table can be built by counting the number of major and minor alleles in both the cases and controls. This leads to the well-known likelihood ratio test for independence, the *G*-test:(5)

where *O_k,h _* is the frequency observed in a cell, and *E_k,h _* is the frequency expected under the null hypothesis in which the allele frequency is the same between cases and controls. The well-known Pearson's chi-square test is asymptotically equivalent to the *G*-test. If the table is generated from true genotypes, then the *G*-statistic asymptotically follows a chi-square distribution with 1 degree of freedom (*χ*^2^(1)). However, in our studies, we construct the *G*-statistic using "called" genotypes, thus HWE may not hold due to over- and under-calling of heterozygotes. Furthermore, constructing the test statistic by counting "called" genotypes instead of "observed" genotypes likely introduces extra variability. Therefore, the statistical theory may not be valid any more. Note that when a genotype is not called for a certain individual, the data is considered missing and is not included in the 2 × 2 table.

### Likelihood ratio test accounting for uncertainty in the observed genotypes for association mapping

Instead of calling genotypes, the likelihood framework allows for uncertainty in the genotypes and tests at each site *j *whether the allele frequency is the same between cases and controls. Formally, we compute the likelihood of the hypotheses *H_O _*: *p_j_*_,1 _= *p_j_*_,2_(= *p_j_*_,0_) and *H_A _*: *p_j_*_,1 _≠ *p_j_*_,2_, where *p_j_*_,1 _and *p_j_*,_2 _are the MAFs in cases and controls, respectively.

Assuming that minor (*m*) and major (*M*) alleles are known, the likelihood of the minor allele frequency can be computed as described in Equation 2, and the likelihood ratio test statistic is computed as:(6)

where  and  are the observed data for cases and controls, respectively, and  and  are the MLEs of the MAFs in cases and controls, respectively.

If the minor allele is unknown, the likelihood under the null hypothesis is computed as in Equation 3, and the *LRT* statistic is modified as:(7)

where *D_j _* is the observed data for both cases and controls, and  is the allele frequency under the null hypothesis. Other notations are the same as in Equation 6.

### Estimating MAF in simulated data

We compare the estimates of allele frequency on simulated data using true genotypes (True), called genotypes without any filtering (Call NF), called genotypes with filtering (*f *= 1; Call F), and the maximum likelihood method (ML). For rare SNPs, the minor allele type is often not apparent. When calling genotypes, the second most common nucleotide is assumed to be the minor allele. The ML method directly incorporates uncertainty in determining the minor allele and unless otherwise stated, results using the unknown minor allele method (Equation 3) are shown. Note that the unknown minor allele ML method performs similarly to the known minor allele ML method but the former better for very rare SNPs (Additional file 1).

**Figure 1:**
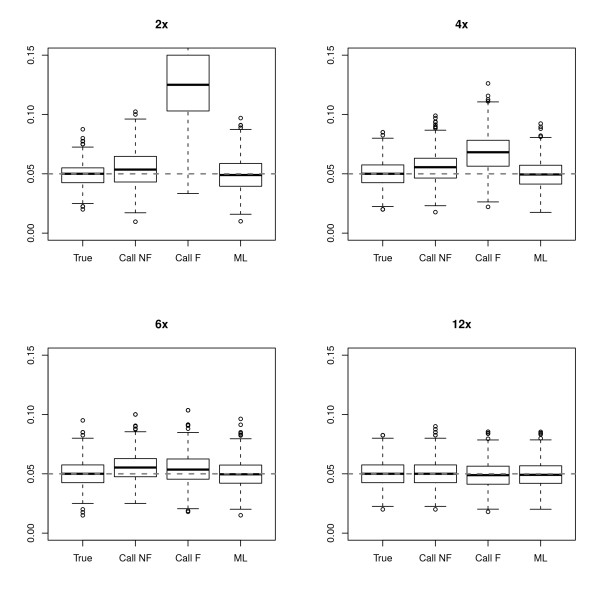
**Estimates of allele frequency at sites with a true MAF of 5% for different depths of coverage**. At each depth, 1,000 sites were simulated using 200 individuals, and at each site, an estimate of allele frequency is computed using: (1) true genotypes (True); (2) called genotypes without filtering (Call NF); (3) called genotypes with filtering (Call F); and (4) the maximum likelihood method (ML). For more details of the estimation methods, see Methods.

We first evaluated how well the different approaches were able to estimate the MAF in 200 individuals across a range of sequencing depths for 1,000 SNPs with a true MAF of 5%. Figure 1 shows boxplots of the distributions of estimated MAFs using the four different approaches. As expected, for higher coverage data, such as an individual depth of 12×, all the methods perform as well as when the genotypes are known with certainty (True). However, when the depth decreases, the estimates of the MAF obtained by first calling genotypes become biased. For example, the median MAF estimated using the Call F method is 5.3% at 6× coverage and is 12.5% at 2×. The reason for the upward bias is that it becomes harder to call heterozygotes since true heterozygotes often look like sequencing errors. Therefore, more heterozygotes than minor homozygotes tend to have missing genotypes. However, the overall bias in MAF estimates from called genotypes is not always in one direction (data not shown). Interestingly, the bias appears to be worse for the Call F method than the Call NF method. This pattern may seem counter-intuitive since filtering the genotype calls would seem to decrease the probability of calling a sequencing error a heterozygote. However, the Call F method also results in a larger amount of missing data since many homozygotes for the major allele will not be called due to sequencing errors. Thus, in this instance, calling genotypes without filtering seems to be the better strategy than filtering genotypes when trying to estimate the MAF.

The results are dramatically different for the new ML method. This method provides unbiased estimates of the MAF (median of ~4.9%) across a range of depths. Even at 2×, the estimates show only a slightly larger variance than those based on the true genotypes.

**Figure 2:**
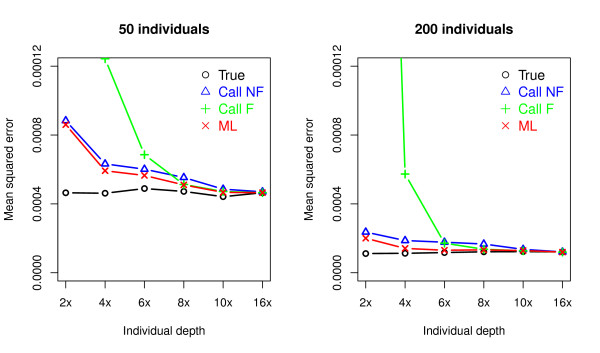
**Mean squred error (MSE; Expected ) of four different types of allele frequency estimators for different sample sizes (left and right panel) and depths of coverage (x-axis)**. At each depth, MSE was computed from the allele frequency estimates made using four different methods: True, Call NF, Call F, and ML (for details of the methods, see the caption of Figure 1).

We also compared the estimated mean squared error (MSE; Expectation () of the different estimates of the MAF across a range of sequencing depths (Figure 2). The ML method has a lower MSE than the calling methods with 50 or 200 individuals. In particular, the MSE computed based on the Call F method is much higher than those from the other methods especially when the depth decreases. The MSE of the estimates of the MAF based on the true genotypes reflects the lower limit of the MSE and is not constant across depths due to sampling variance and a finite sample size. Using 50 individuals, the MSE approaches 0.0005 with increasing depth and when using a sample size of 200 individuals, it approaches 0.0013 with increasing depth.

Overall, the new ML method out-performs genotype calling methods.

### Estimating a distribution of MAFs from simulated data

**Figure 3:**
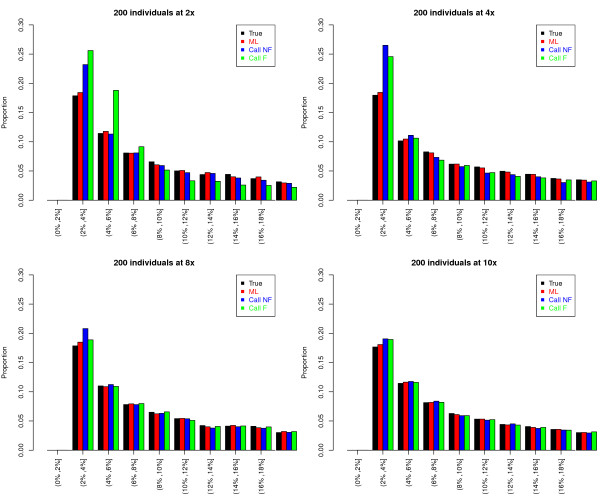
**Distribution of allele frequencies of SNPs simulated assuming the standard stationary distribution of allele frequencies**. At each depth (each panel), 20,000 SNPs were simulated, and for each SNP, estimates of the MAF were obtained using four different methods (see the caption of Figure 1). Then, for each method (each color), only sites with estimated allele frequencies > 2% are used to generate each histogram (x-axis).

We next examine how the different estimation approaches performed in estimating the proportion of SNPs at different frequencies in the population (similar to the site frequency spectrum but based on population allele frequency instead of sample frequency). Here we simulated 20,000 SNPs where the distribution of the true MAFs followed the standard stationary distribution for an effective population size of 10,000 (see Methods). Note that in practice, however, it is very difficult to distinguish a very rare SNP from a sequencing error. Therefore, for comparison purpose with real data, we discarded SNPs with estimated MAF less than 2%. Figure 3 shows the proportion of SNPs falling into each different frequency bin after excluding those SNPs with estimated MAF<2%.

As expected, with a high depth of coverage, such as 10× per individual, all methods provide estimated MAF distributions that are similar to the expected distribution based on the true genotypes (Figure 3). With a shallower depth of coverage, such as less than 4× per individual, the distributions of MAFs obtained by genotype calling methods significantly depart from the expected MAF distribution based on true genotypes (Figure 3). In particular, these methods over-estimate the proportion of low-frequency SNPs. For example, the expected proportion of SNPs in the second bin (estimated MAF between 2-4%) is 18%. The corresponding proportion based on the Call NF method at a depth of 4× is 26%, which is 1.4-fold higher than expected. The over-estimation of the proportion of low-frequency SNPs occurs due to confusion of sequencing errors with true heterozygotes, which results in overcalling heterozygous genotypes. The magnitude of this inflation differs across different filtering cutoffs, but a larger cutoff does not necessarily increase or decrease the inflation.

The picture is entirely different for the ML method. The estimated MAF distribution obtained from the new ML method closely follows the true distribution even with shallow depths of coverage. Here there is almost no excess of low-frequency SNPs. At a depth of 4×, the proportion of SNPs in the second bin of the histogram is 18.4%, which is very close to the expected proportion (18%). Thus, more reliable estimates of the frequency spectrum can be made from low-coverage data by using our likelihood approach than by using the genotype calling approaches.

### Association mapping in simulated data

We compare the performance of methods that treat inferred genotypes as true genotypes in tests of association (using a *G*-test) to our likelihood ratio test (LRT) that accounts for uncertainty in the genotypes. We examine the distribution of the test-statistic under the null hypothesis of no allele frequency difference between cases and controls. We also compare the power of the different approaches.

**Figure 4:**
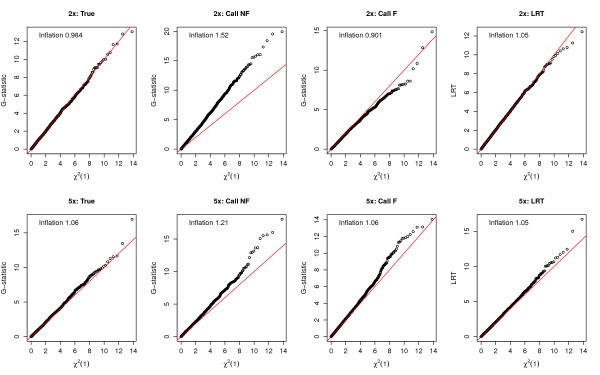
**QQ-plots comparing the null distribution of the test statistic of interest with a *χ*^2^(1) distribution**. Each column corresponds to a different test statistic: (1) *G*-statistic computed using the true genotypes (True); (2) *G*-statistic computed using called genotypes without filtering (Call NF); (3) *G*-statistic computed using called genotypes with filtering (Call F); and (4) the likelihood ratio test statistic with unknown minor allele (LRT). Assuming 500 cases and 500 controls, under the null hypothesis, a set of 5,000 sites were simulated with a MAF of 5% with a sequencing depth of 2× (upper panels) and 5× (lower panels). The "Inflation" factor [44] is shown in the upper left corner of each figure.

With reasonably large sample sizes, standard asymptotic theory suggests that under the null hypothesis both the *G*-statistic and *LRT *statistic follow a chi-square distribution with one degree of freedom (*χ*^2^(1)). Therefore, we have compared the null distribution of the *G*-statistic computed based on calling methods as well as the *LRT *statistic to the *χ*^2^(1) distribution using QQ-plots (Figure 4). We simulated 5,000 SNPs across a variety of sequencing depths in 500 cases and controls where the MAF used to simulate genotypes was 5% in both cases and controls. The distribution of the *G*-statistic computed using the true genotypes shows a very good correspondence with a *χ*^2^(1) distribution. However, the distribution of the *G*-statistic computed based on the called genotypes substantially departs from a *χ*^2^(1) distribution. Calling genotypes and then treating those genotypes as being accurate produces a vast excess of false-positive signals if the *p*-values are computed using a *χ*^2^(1) distribution. For example, at a depth of 2×, 11% of the SNPs had a *p*-value less than 5%, compared to the expected 5%. The effect is caused by an increase in the variance, due to overcalling homozygotes as heterozygotes, in the allelic test used here for detecting association. Genotypic tests such as Armitage trend test, which are robust to deviations from Hardy-Weinberg equilibrium, do not show a similar increase in the false positive rate (Additional file 2). Consistent with this observation, filtering the called genotypes results in a decrease in the fraction of significant tests when using the *G*-test, although filtering does not completely solve the problem. On the other hand, the *LRT *statistic shows only a very slight departure from a *χ*^2^(1) distribution for either 2× or 5× depths of coverage.

**Figure 5:**
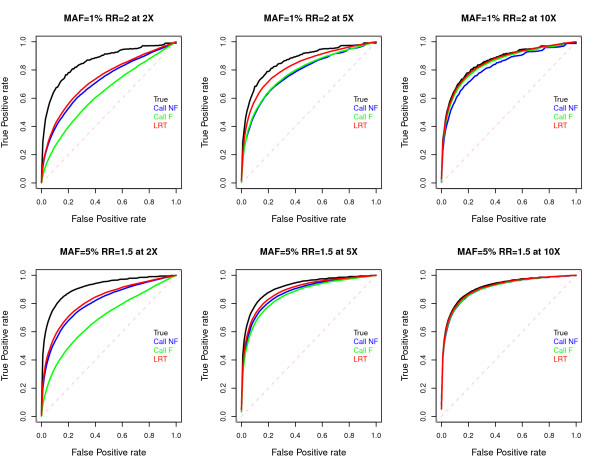
**Receiver operating characteristic (ROC) curves of four tests of association**. For the definition of the four statistics, see the caption of Figure 4. Assuming 500 cases and 500 controls, a set of 20,000 sites were simulated under the null and under the alternative at individual sequencing depths of 2×, 5×, and 10× (three columns). At each false positive rate (x-axis), the corresponding critical value was computed using the empirical null distribution. The true positive rate (power; y-axis) was obtained by computing the fraction of causative sites with test statistics that exceed the critical value.

We also generated receiver operating characteristic (ROC) curves for each of the different association tests. These curves show the power of the test at different false-positive rates. Since the distributions of some of the test statistics do not follow the *χ*^2^(1) distribution under the null hypothesis, to make a fair comparison, we obtained the critical value for each false positive rate based on the empirical null distribution. The power is computed as the fraction of simulated disease loci that have a statistic exceeding the critical value. Overall, we find that the LRT* *performs better than the *G*-test based on either genotype calling method (Figure 5). For example, at a 5% false positive rate and with a sequencing depth of 5×, the power to detect a disease locus with a MAF of 1% and a relative risk (RR) of 2 is 51% with the LRT, but power drops to 33% using the calling method without filtering and to 34% using the calling method with filtering. In particular, at low depth, the *G*-test applied to called genotypes with filtering performs very poorly (left most column in Figure 5). If we compare the power of the *LRT *to the Armitage trend test using called genotypes, we find that the LRT also has higher power than the Armitage trend test (Additional file 3). This suggests that if one wishes to use called genotypes, filtering them based on call confidence can result in a loss of power.

### Application to real data

We analyzed 200 exomes from controls for a disease association study that have been sequenced using Illumina technology at a per-individual depth of 8× [42]. We used the genotype likelihoods generated by the "SOAPsnp" program [32] for our inference. For more details, see Methods.

**Figure 6:**
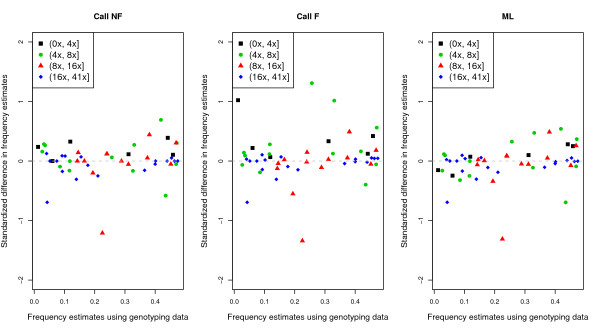
**Estimates of allele frequency computed from 200 individuals using next-generation sequencing data vs. Sequenom genotype data**. At each site, only individuals that have both Sequenom genotype data and sequencing data were used for estimation of allele frequency. For the sequencing data, estimates of MAF were obtained using three different methods (Call NF; Call F; and ML). The standardized difference for each estimate was computed as , where  and  are the estimated MAFs from the sequencing data and Sequenom genotype data, respectively, and *n *is the number of individuals used for the estimation. Each site is classified into one of four bins based on average individual depth of coverage (color): less than 4×, higher than 4× but less than 8×, higher than 8× but less than 16×, and higher than 16×.

First, we explored the accuracy of the estimates of the MAF from next-generation sequencing data for 50 SNPs by comparing them to the estimated MAFs from Sequenom genotype data. Both the estimates using the ML method and the genotype calling method without filtering are highly correlated with the estimates made from the Sequenom genotype data (i.e., a small standardized difference between the two estimates in Figure 6). However, estimates based on genotype calling with filtering show poor correspondence to the frequencies estimated from the Sequenom genotype data, especially when sequencing depth is low. Interestingly, there is one SNP where the estimated MAF from the resequencing data is very different from the estimate obtained from the Sequenom genotype data, even though the sequencing depth is very high (14×). Specifically, the estimated MAF from the Sequenom genotype data is 22.5%, but is 17.2% when estimated using the ML approach. Individual examination shows that in many individuals, the highly supported genotype based on the sequencing data differs from the Sequenom genotypes. Given that this SNP is covered by many reads in these individuals and that the observed read bases have high quality scores (>Q20), it is likely that the difference is due to Sequenom genotyping errors. Note that there are a couple of SNPs in which the estimated MAFs from the genotype calling approach without filtering seem to better correspond to the MAFs estimated from the Sequenom genotyping than the estimates from the ML approach do. For example, at one SNP the estimated MAF is 25.7% from the Sequenom genotype data, 25.9% from the genotype calling method without filtering, and 27.2% from the ML method. However, individual inspection reveals there are a few individuals for which the called genotype from the sequencing data differs from the Sequenom genotype. In these cases, the errors in the called genotypes canceled, giving the appearance of better correspondence with the Sequenom genotype data. Therefore, for these SNPs, it is hard to tell which method performs best.

**Figure 7:**
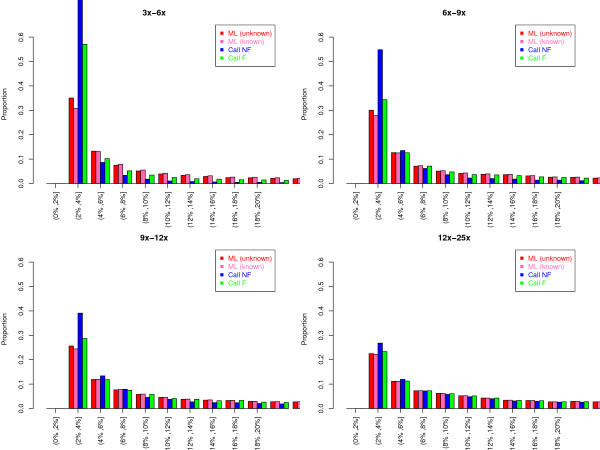
**Distribution of the minor allele frequency estimated from the exomes of 200 sequenced individuals**. For each site, the minor allele frequency was estimated using four different methods: (1) the ML method with unknown minor allele, (2) the ML method with a known or fixed minor allele, (3) calling genotypes without filtering (Call NF), and (4) calling genotypes with filtering (Call F). Each site is classified into bins based on the depth of coverage. Furthermore, in each histogram, sites with estimated MAF less than 2% are not considered. For the number of SNPs that were used for this analysis, see Table 1.

We next examined the distribution of MAFs computed using several approaches across a range of sequencing depths from our next-generation exome sequencing data (Figure 7). We discarded SNPs with estimated MAF <2% since it is difficult to distinguish these very low-frequency SNPs from sequencing errors in this dataset. We further removed sites in which there was a significant difference (*p*-value less than 10^-5 ^using a rank-sum-test [43]) in the quality score of read bases between the minor and major alleles. These sites are likely to be artificial SNPs that may occur due to incorrect mapping or unknown biases introduced during the experimental procedure. Then we classified each site into bins based on the depth of coverage. The number of SNPs in each bin is shown in Table 1. When the average depth is less than 9×, the distributions of estimated MAFs based on genotype calling methods are very different from the one based on the ML method. Specifically, the genotype calling approaches give rise to a large excess of low-frequency SNPs (MAF between 2% and 4%). This pattern mirrors what was seen in our simulation studies (Figure 3). Also, for the genotype calling methods, the allele frequency distribution changes dramatically as sequencing depth changes. Therefore, as discussed previously, when depth is not very high, the genotyping calling methods are likely to include a lot of false SNPs that are sequencing errors. These errors appear as an excess of low-frequency SNPs in the frequency distribution. The distribution based on the ML method is more stable across depths, but there still is an excess of SNPs with low allele frequency with depth less than 9×as compared to the proportion of low-frequency SNPs at greater depths.

**Table 1:** Number of SNPs with estimated MAF larger than 2% using a particular method (row) within each bin (column) defined by average sequence depth across individuals.

	0.5×-3×	3×-6×	6×-9×	9×-12×	12×-25×
ML (unknown)	18324	12564	9102	6778	11862
ML (known)	15282	11482	8742	6651	11810
Call NF	123546	63415	19516	9695	13035
Call F	391488	21511	10018	7145	12026

**Figure 8:**
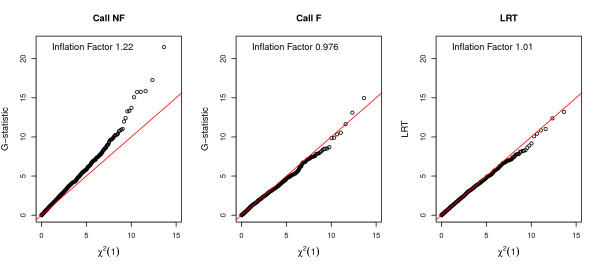
**QQ-plots comparing the association test statistics for allele frequency differences between 100 cases and 100 controls to a *χ*^2^(1) distribution**. Phenotypes were randomly assigned to indivdiduals in the exome resequencing dataset such that there are 100 cases and 100 controls. For each site, three statistics were computed: the *G*-statistic using called genotypes without filtering (Call NF), the *G*-static using called genotypes with filtering (Call F), and the *LRT *statistic. To minimize inclusion of false SNPs, sites with ML MAF estimates less than 2% are discarded. For display purposes, results from sites on chromosome 2 are shown. Note that the inflation factor is shown in the upper left corner of each QQ-plot.

Finally, we used this exome-resequencing data to simulate a case-control association study. To examine the distribution of the association test statistics under the null hypothesis, we randomly assigned 100 individuals to a case group and the other 100 to the control group. For all SNPs on chromosome 2 with MAF estimates > 2% (based on the unknown minor allele ML method), we tested for allele frequency differences between cases and controls by computing the *G*-statistic using called genotypes both with and without filtering as well as the *LRT *statistic. Figure 8 shows the QQ plots comparing the distributions of the test statistics to the standard *χ*^2^(1) distribution. As seen in simulation studies, the null distribution of the *G*-statistic calculated when calling genotypes without filtering substantially departs from the *χ*^2^(1) distribution. However, the null distribution of the *LRT *statistic closely follows the *χ*^2^(1) distribution. The inflation factor [44] is 1.01, implying that *LRT *statistic performs well when applied to real data.

## Discussion

The likelihood method discussed here is an extension of our previous approach [30] which was similar to that of Lynch [29]. We have improved this approach by allowing for uncertainty in determining which allele is the minor allele. Additionally, the present formulation includes base-specific error rates (see Equation 8). These additions may have a practical benefit particularly when estimating the frequencies of rarer alleles, where it may not be obvious which allele is the minor allele and where sequencing errors may have the greatest effect on frequency estimation.

Though not surprising, it is important to note that with higher sequencing coverage, the particular approach used to estimate allele frequencies does not matter as much. For depths of coverage > 10×, the genotype calling methods both with and without filtering behave appropriately and similarly to the ML approach. Thus, with high depths of coverage, the traditional and simple method of calling genotypes and then treating those genotypes as being known with certainty is still effective. The reason for this is that with such high depth, the called genotypes are likely to be accurate. With lower depths of coverage, however, there is considerable uncertainty regarding the true genotype. Often the most-likely genotype will not be the true genotype, leading to biases in estimates of allele frequency and spurious signals of association in case-control studies. In this situation, the ML method is a superior approach.

In our simulations, we compared the performance of our ML approach to a relatively simple genotype calling approach (see Methods). It is possible that more sophisticated genotype calling approaches such as SOAPsnp [32], MAQ [23], and GATK [45] may show improved performance relative to the simple genotype calling approach used here. However, many of the same trends found in our simulations, where the simple genotype calling approach was used, were also seen in the exome sequencing data where genotypes were called using SOAPsnp. For example in both the simulations and the exome data, genotype calling approaches result in the appearance of an excess of low-frequency SNPs (compare Figure 3 to Figure 7) especially when sequencing depth is less than 8×per individual.

We have explored whether it is better to call genotypes with filtering or without filtering when analyzing low-coverage data. Intuitively, one would expect that if there was uncertainty in the genotypes, it would be better to call genotypes only if one was very confident in that genotype and treat the other less confident genotypes as missing data. However, as discussed by Johnson et al. [46], calling genotypes only when one is very confident can adversely affect downstream analyses that use the inferred genotypes. Our simulations and analyses of real data show that for estimating allele frequencies, genotype calling methods perform better without any filtering because filtering creates a strong upward bias in the frequency estimates. For association studies, it is not always clear whether it is better to filter the genotypes. Not filtering can result in an excess of false-positive results for allelic-based tests, but filtering can result in a decrease in power.

Studies have suggested that genotype calling approaches that use LD information to call genotypes [21,36] may result in more accurate inferences from low-coverage data. However, it is unclear whether using population genetic characteristics of the data, like LD patterns, to call genotypes biases downstream population genetic and evolutionary analyses. Such an evaluation is beyond the scope of the present work. However, this is not a concern for our method to estimate allele frequencies because our approach does not use any LD information.

As currently implemented our method does not tackle the problem of SNP calling itself. In principle, our approach could be extended to use a LRT to call SNPs. Specifically, the test could compare the probability of the data under the hypothesis that there is no SNP at a given site (*H*_0 _: *p *= 0) and under the hypothesis that there is a SNP at a given site (*H_A _*: *p *> 0). Such an approach is the subject of ongoing research. A feature of our approach to estimating frequencies is that it is not necessary to first call SNPs prior to estimating frequencies, which may be beneficial in certain circumstances.

Finally, our likelihood method has some limitations. It cannot estimate the frequencies of very rare alleles from low-coverage data. This is not so much a deficiency with the likelihood approach, but instead, speaks to the difficulty in detecting very rare variants using little data in a background of sequencing errors. To reliably detect and estimate the frequencies of rare variants with < 1% frequency, higher-coverage sequencing will be required. However, approaches that take genotype uncertainty into account may still be important. As shown by Garner [47], not taking uncertainty into account can lead to an increased false positive rate in association studies when sequencing depth varies between cases and controls. Our approach also assumes that the loci analyzed are in Hardy-Weinberg equilibrium. While this is a reasonable assumption for most human populations, our approach can be extended to consider more complex relationships between allele frequencies and genotype frequencies. We want to emphasize that all methods to estimate allele frequency from next-generation sequencing data require that accurate genotype likelihoods can be calculated. If these calculations are not accurate, even after recalibration, none of the methods are likely to perform well.

## Conclusions

We have evaluated the performance of a likelihood method and genotype calling methods to estimate the minor allele frequency from next-generation sequencing data. The likelihood method accurately estimates allele frequencies even when applied to low-coverage data (e.g., < 4×per individual) since it models the uncertainty in assigning individual genotypes. However, genotype calling approaches can lead to biased inferences when applied to low-coverage data. We have also extended the likelihood approach to test for differences in estimated minor allele frequency between cases and controls. Through simulations and the analysis of exomes from 200 individuals, our LRT has appropriate false-positive rates and higher power than genotype calling approaches when analyzing low-coverage data. Finally, we have shown that under certain circumstances, if one uses genotype calling approaches, it is better to not filter genotypes based on the call confidence score.

## Methods

### Simulation studies

We performed extensive simulation studies to compare the performance of the likelihood methods with methods based on called genotypes. Specifically, we simulated data to assess (1) the accuracy of the estimates of the MAF, (2) the accuracy in estimating the distribution of MAFs across genome, and (3) statistical power in association mapping studies. Due to computational constraints, we simulated SNPs in the sequencing data directly rather than simulating raw sequencing reads.

We simulated SNPs with a specified MAF, number of individuals and per-individual sequencing depth. When simulating causal SNPs in association studies, MAFs for cases and controls were assigned using a multiplicative disease model. For this model, the prevalence of the disease was fixed at 10%. We examined two sets of MAFs and relative risks. First, the combined MAF in cases and controls was 1% and the relative risk was 2. Second, the combined MAF was 5% and the relative risk was 1.5. As an example, with a combined MAF of 1% and a relative risk of 2.0, the obtained MAFs for cases and controls are 1.98% and 0.89%, respectively. Each individual genotype was simulated assuming Hardy-Weinberg equilibrium with the given MAF. Read bases were then generated for each individual by copying each allele a Poisson-distributed number of times with mean equal to the half the specified individual depth. Each read base then may have been altered to one of the other three nucleotides at a specified type-specific sequencing error rate. The type-specific error rates used to simulate the data were estimated (see below) from 200 exomes sequenced using the Illumina platform [42]. The simulation output was summarized as the number of reads for each of the four nucleotides (A,C,G,T) for each individual.

We also evaluated the performance of the approaches to estimate a distribution of MAFs when the specified allele frequencies were drawn from a the stationary distribution under a Wright-Fisher model with a population size of 10,000. Under such a model, population allele frequencies are proportional to 1/x, where x is the frequency of the allele in the population [48]. Given the population allele frequency for each site, genotypes and read counts were simulated as described above.

Various methods have been proposed to compute genotype likelihoods from next-generation sequencing data, which recalibrate quality scores of read bases and attempt to correct for sequencing error structures and other complexities in the genome [23,32]. However, for practical reasons, our simulation studies use a simple genotype likelihood mainly based on the number of reads for each base and globally-determined base-specific sequencing error rates. This model is described below.

The data consist of the observed counts of each of the four nucleotides (*nA_i_*,*_j_*, *nC_i_*,*_j_*, *nG_i_*,*_j_*, *nT_i_*,*_j_*) in each individual *i *at site *j*. Denote the twelve base-specific sequencing error rates as , where *ε_b_*,*_b' _* = *P*(read = *b'*|allele = *b*), where *b*, *b' *∈ {*A*, *C*, *G*, *T*} and *b *≠ *b*. Given these error rates, the likelihood for genotype *g*(∈ {0, 1, 2}) is computed as follows:(8)

where *m*, *M*, *o*1 and *o*2 denote for the minor allele, major allele, and other two nucleotides, respectively (the order of other two nucleotides does not matter). Note that the counts of four nucleotides are re-ordered as counts of minor, major, and other two nucleotides (*nm_i_*,*_j_*, *nM_i_*,*_j_*, *no*1*_i_*,*_j_*, *no*2*_i_*,*_j_*), and the set of error rates is also re-arranged to , where *h*, *h' *∈ {*m*, *M*, *o*1, *o*2} for each site. These genotype likelihoods were computed from our simulated datasets. The globally-determined base-specific sequencing error rates were set to the true values used to simulate the data (estimated from 200 exome sequencing data [42]; see Additional file 4), since in practice 10,000 sites were enough to obtain stable estimates of the type-specific error rates.

### Analysis of of real data

We also analyzed 200 Danish exomes that had been sequenced using Illumina technology at a coverage of about 8×per individual [42]. These individuals were controls selected for a disease mapping study. We used the genotype likelihoods generated by the "SOAPsnp" program [32] for our inference.

We examined the performance of the ML approach as applied to this dataset in three different ways. First, we used 50 SNPs in which Sequenom genotype data were available in most of individuals to compare the MAFs estimated from the Illumina sequencing data to those estimated from the genotype data. Here, for each site, we used only those individuals that have both genotype data and sequence data. For most of the sites (>95%), more than 170 individuals satisfy this condition. Second, we examined the proportion of SNPs with different frequencies when using different strategies to estimate the MAFs. Finally, we used these data to simulate a case-control association study by randomly assigning 100 exomes to a case group. We then examined the behavior of the different test statistics under the null hypothesis.

## Availability of software

All the source code used for our simulation studies, estimation of parameters, and tests of association are publicly available (Additional files 5 and 6).

## Authors' contributions

SYK participated in the design of the study, carried out simulation studies and statistical analyses, and was involved in drafting the manuscript. KEL was involved in drafting the manuscript and revising it critically for important intellectual content. AA and TSK participated in processing the sequence data and helped with computational aspects of the project. YL, GT, NG, TJ, GA, DW, TJ, TH, OP and JW participated in the cohort design and data coordination. RN conceived of the study, and participated in its design and coordination and helped to draft the manuscript. All authors read and approved the final manuscript.

## Supplementary Material

Additional file 1 **Boxplot of estimated MAFs using ML methods with known or unknown minor allele**. Boxplot of estimated MAFs of SNPs corresponding to each sample allele frequency. Assuming 1,000 individuals, 1,000 SNPs with true MAF of 0.5% were simulated at individual sequencing depth of 8*X*. For each SNP, sample allele frequency was obtained using true genotypes (x-axis). Then each boxplot was drawn using estimated MAFs with known (left) and unknown(right) minor alleles. Click here for file

Additional file 2 **QQ-plot comparing the null distribution of the Armitage trend test statistic with a *χ* ^2^(1) distribution**. QQ-plots comparing the null distribution of the test statistic of interest with a *χ*^2^(1) distribution. The first three columns correspond to the Armitage trend test statistic computed using the true genotypes (True), called genotypes without filtering (Call NF), and called genotypes with filtering (Call F), respectively. The fourth column corresponds to the likelihood ratio test statistic with unknown minor allele (LRT). Assuming 500 cases and 500 controls, under the null hypothesis, a set of 5,000 sites were simulated with a MAF of 5% with a sequencing depth of 2× (upper panels) and 5× (lower panels). The "Inflation" factor [44] is shown in the upper left corner of each figure. Click here for file

Additional file 3 **Receiver operating characteristic curve of the Armitage trend test**. Receiver operating characteristic (ROC) curves of four tests of association. For the definition of the four statistics, see the caption of Additional file 2. Assuming 500 cases and 500 controls, a set of 20,000 sites were simulated under the null and under the alternative at individual sequencing depths of 2×, 5×, and 10× (three columns). At each false positive rate (x-axis), the corresponding critical value was computed using the empirical null distribution. The true positive rate (power; y-axis) was obtained by computing the fraction of causative sites with test statistics that exceed the critical value. Click here for file

Additional file 4 **Estimates of type-specific sequencing error rates**. Type-specific sequencing error rates estimated from 200 exomes [42] using our models (Equation 8). Click here for file

Additional file 5 **Manual of our programs: ****simreseq**** and ****testassoc**. Manual of our programs: simreseq and testassoc. Click here for file

Additional file 6 **Source code of our programs: ****simreseq ****and ****testassoc**. All the source code of our programs used for the simulation studies, estimation of parameters, and tests of association. Click here for file
