# Supplementary figures and images for: Estimation of allele frequency and association mapping using next-generation sequencing data
Source: BMC Bioinformatics. 2011 Jun 11;12:231. doi: 10.1186/1471-2105-12-231 (PMC3212839; doi:10.1186/1471-2105-12-231)

**ML (known)**

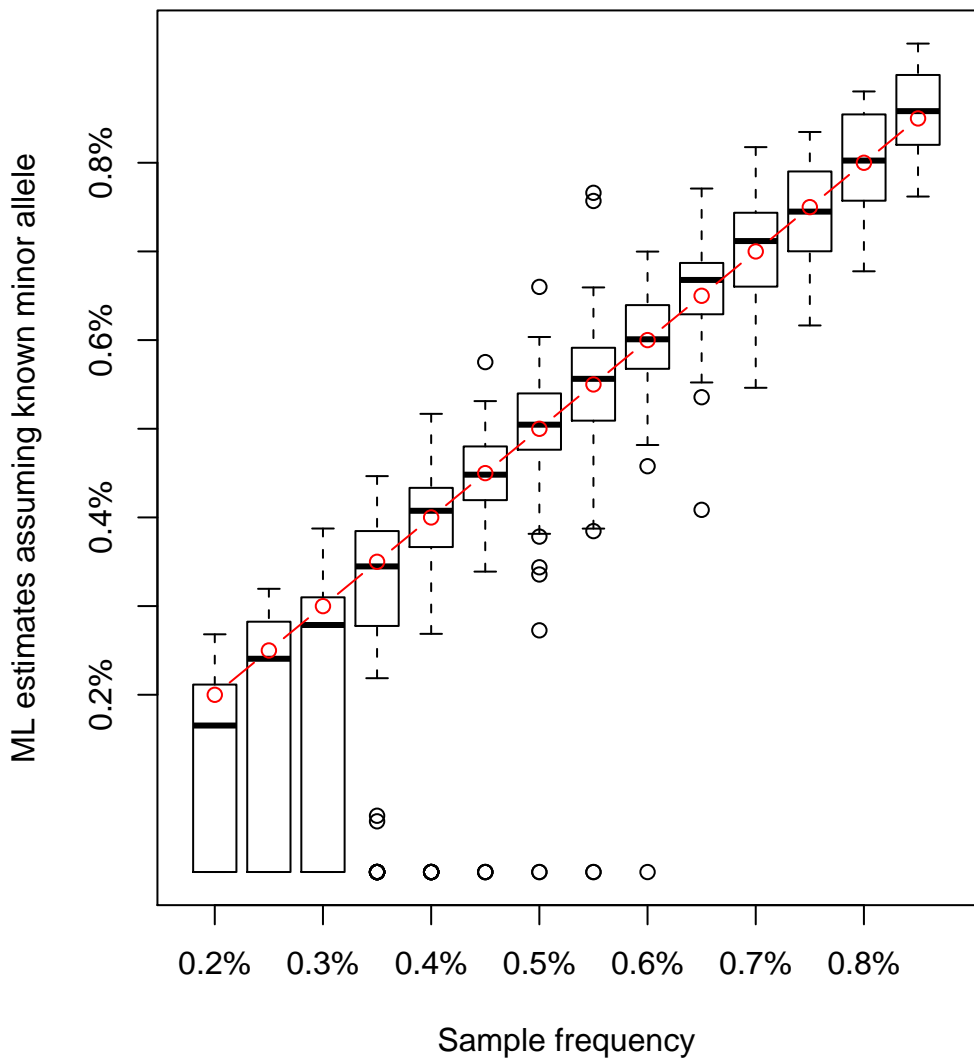

**ML (unknown)**

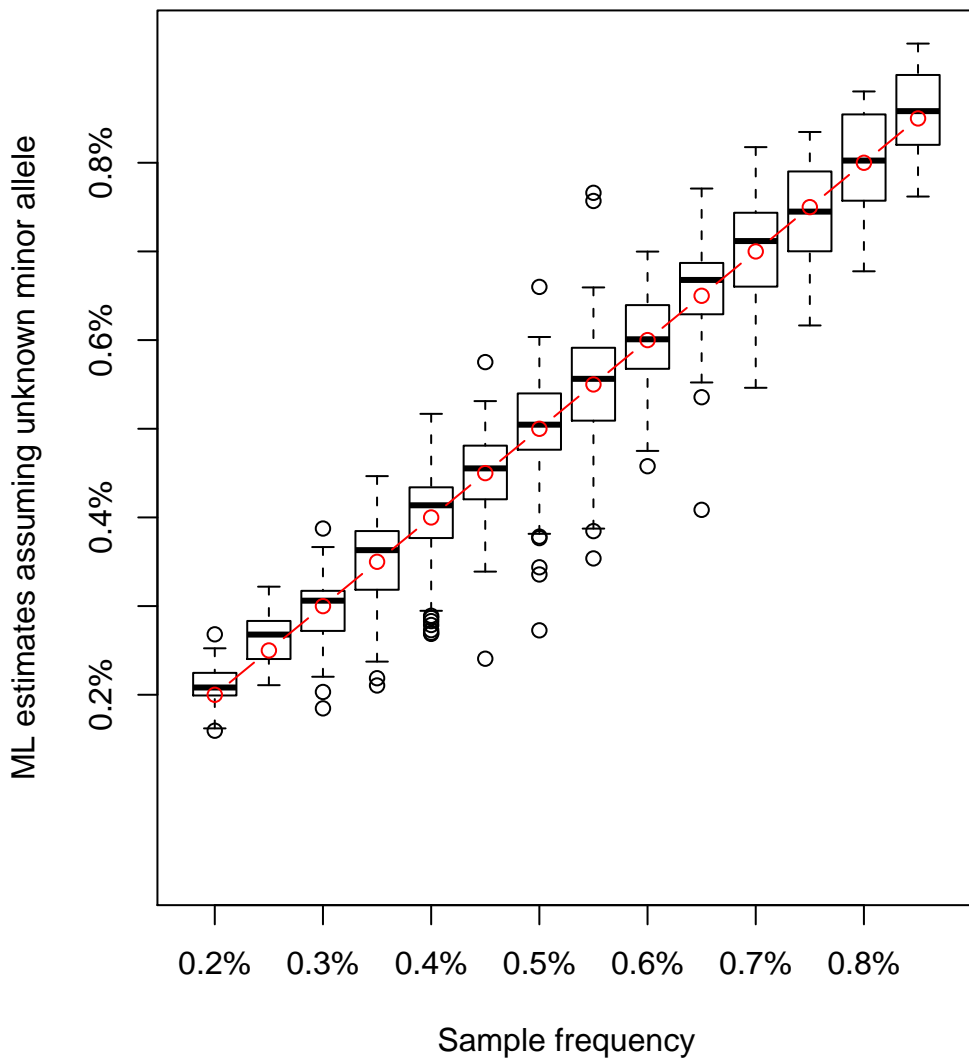

Supplement: Additional file 1 — Boxplot of estimated MAFs using ML methods with known or unknown minor allele. Boxplot of estimated MAFs of SNPs corresponding to each sample allele frequency. Assuming 1,000 individuals, 1,000 SNPs with true MAF of 0.5% were simulated at individual sequencing depth of 8X. For each SNP, sample allele frequency was obtained using true genotypes (x-axis). Then each boxplot was drawn using estimated MAFs with known (left) and unknown(right) minor alleles. [file 1471-2105-12-231-S1.PDF]

**2x: True**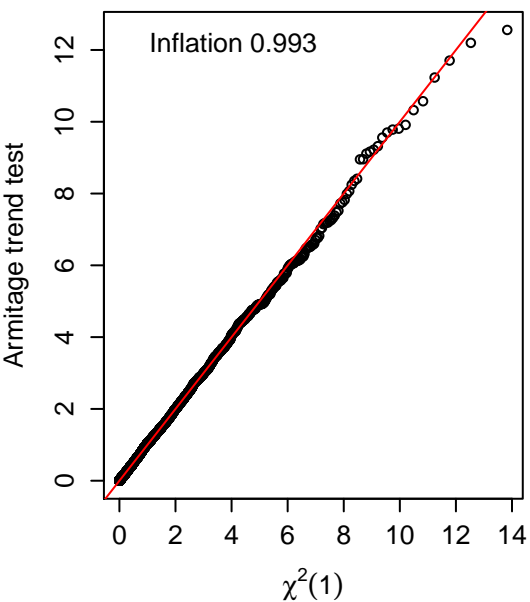**2x: Call NF**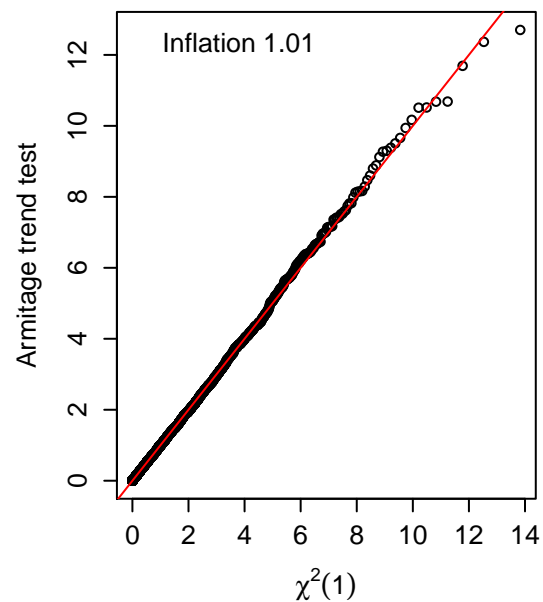**2x: Call F**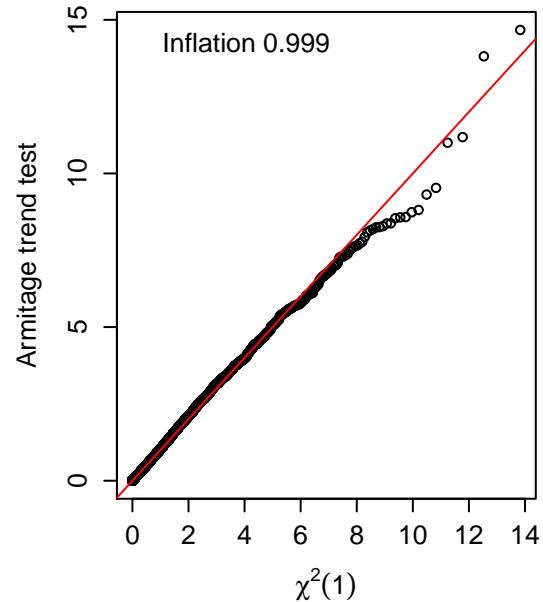**2x: LRT**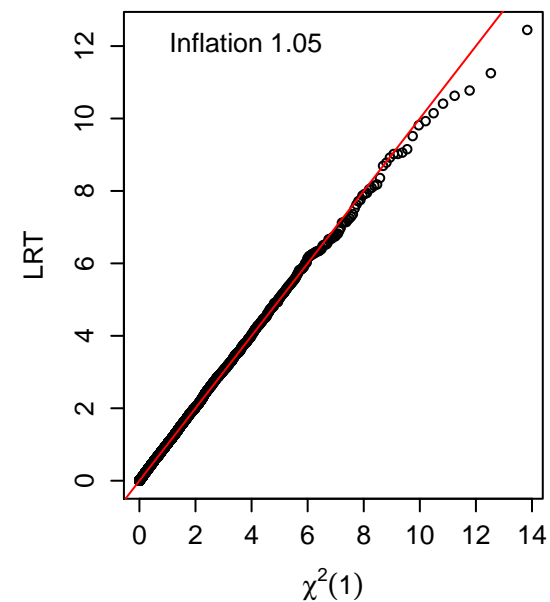**5x: True**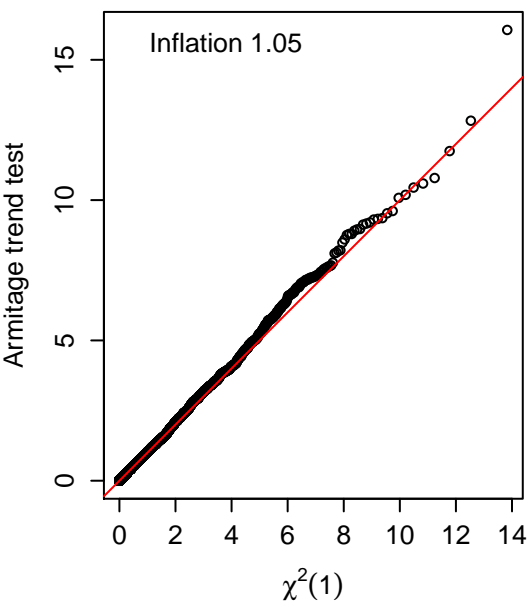**5x: Call NF**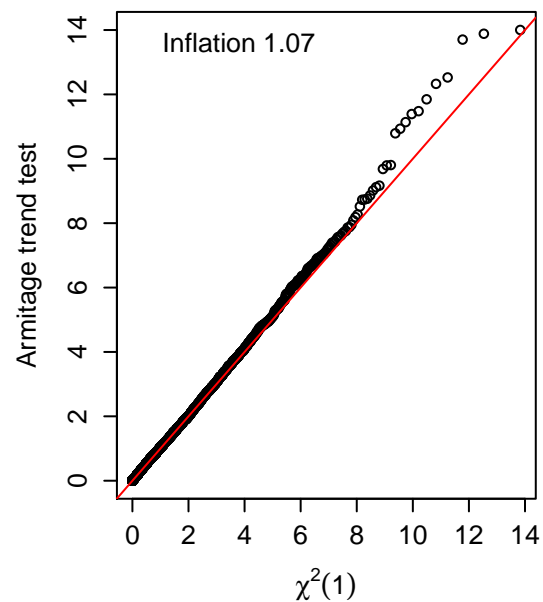**5x: Call F**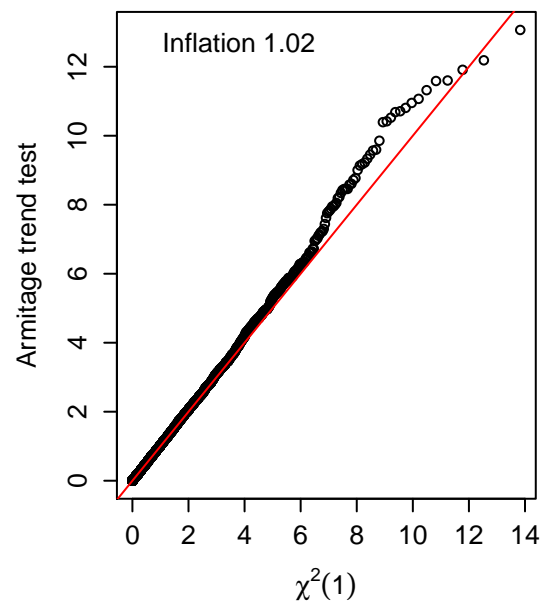**5x: LRT**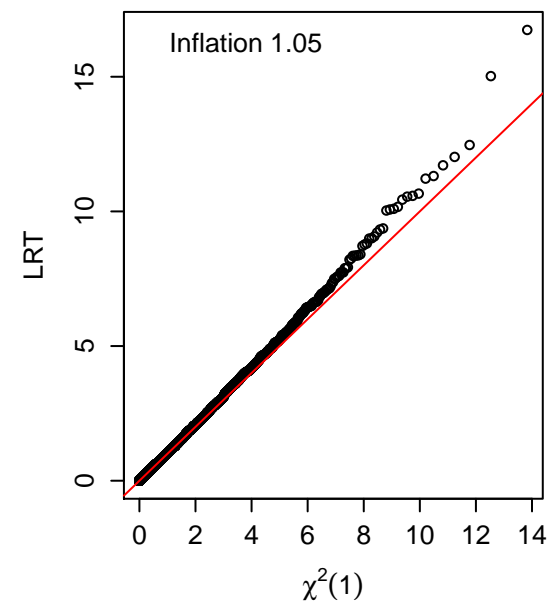

Supplement: Additional file 2 — QQ-plot comparing the null distribution of the Armitage trend test statistic with a χ 2(1) distribution. QQ-plots comparing the null distribution of the test statistic of interest with a χ2(1) distribution. The first three columns correspond to the Armitage trend test statistic computed using the true genotypes (True), called genotypes without filtering (Call NF), and called genotypes with filtering (Call F), respectively. The fourth column corresponds to the likelihood ratio test statistic with unknown minor allele (LRT). Assuming 500 cases and 500 controls, under the null hypothesis, a set of 5,000 sites were simulated with a MAF of 5% with a sequencing depth of 2× (upper panels) and 5× (lower panels). The "Inflation" factor [44] is shown in the upper left corner of each figure. [file 1471-2105-12-231-S2.PDF]

**MAF=1% RR=2 at 2X**

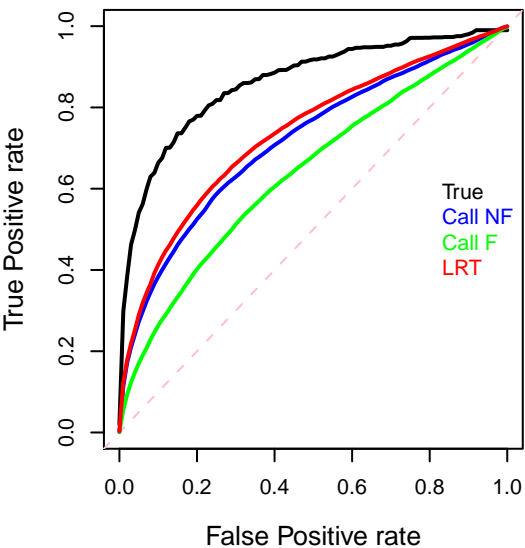

**MAF=1% RR=2 at 5X**

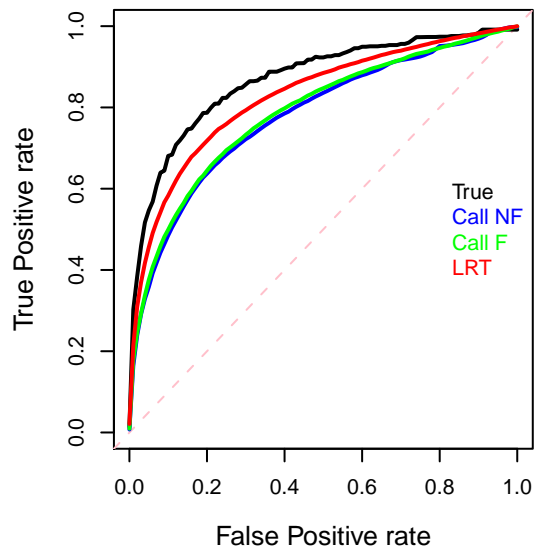

**MAF=1% RR=2 at 10X**

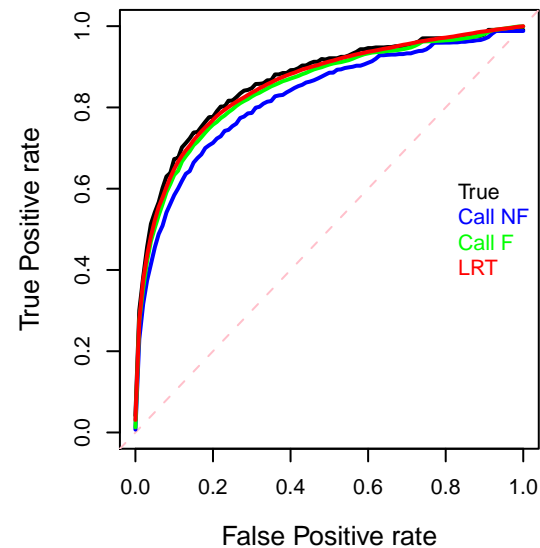

**MAF=5% RR=1.5 at 2X**

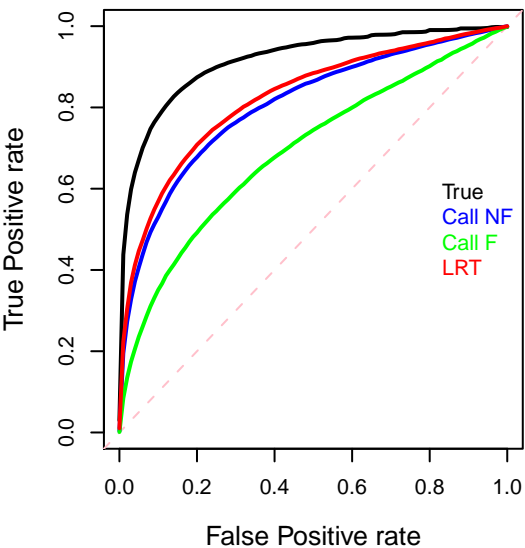

**MAF=5% RR=1.5 at 5X**

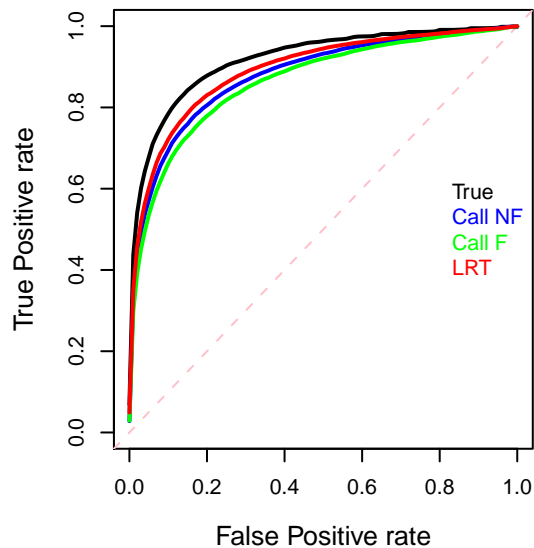

**MAF=5% RR=1.5 at 10X**

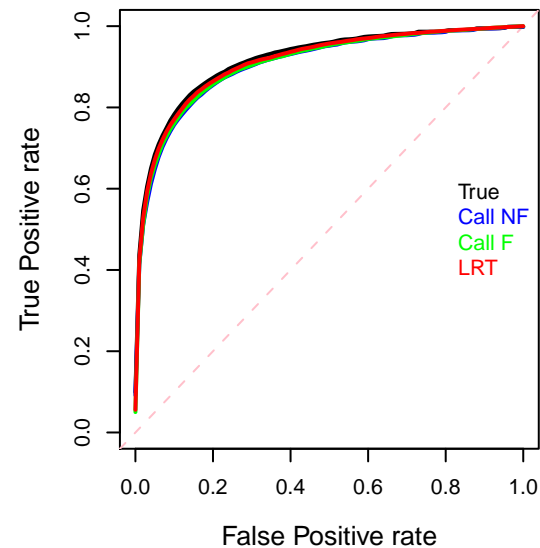

Supplement: Additional file 3 — Receiver operating characteristic curve of the Armitage trend test. Receiver operating characteristic (ROC) curves of four tests of association. For the definition of the four statistics, see the caption of Additional file 2. Assuming 500 cases and 500 controls, a set of 20,000 sites were simulated under the null and under the alternative at individual sequencing depths of 2×, 5×, and 10× (three columns). At each false positive rate (x-axis), the corresponding critical value was computed using the empirical null distribution. The true positive rate (power; y-axis) was obtained by computing the fraction of causative sites with test statistics that exceed the critical value. [file 1471-2105-12-231-S3.PDF]

**Estimates of type-specific error rates**

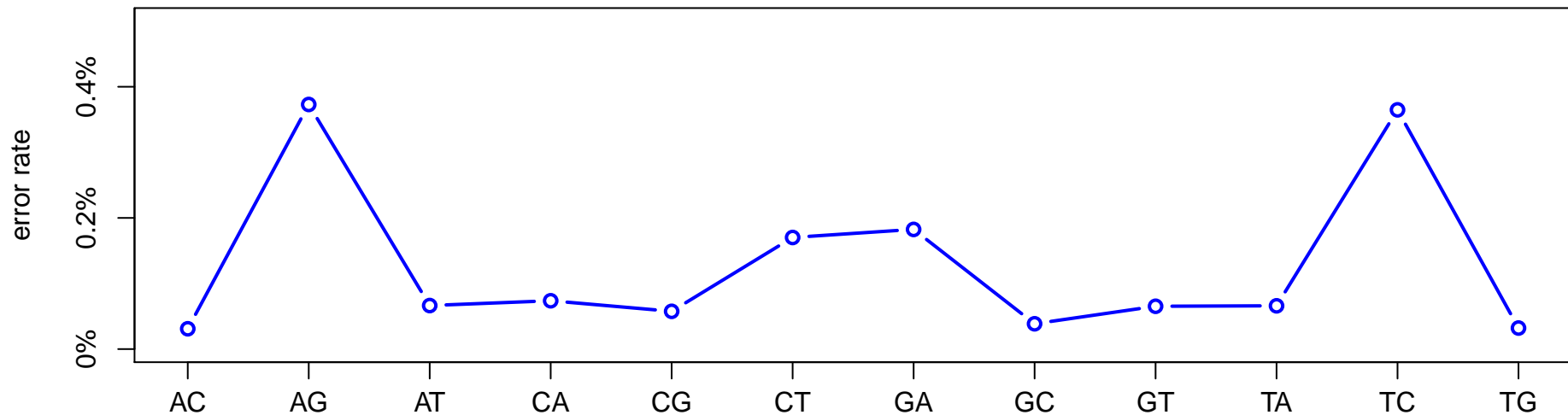

Supplement: Additional file 4 — Estimates of type-specific sequencing error rates. Type-specific sequencing error rates estimated from 200 exomes [42] using our models (Equation 8). [file 1471-2105-12-231-S4.PDF]
