# Supplementary material for: Estimation of allele frequency and association mapping using next-generation sequencing data
Source: BMC Bioinformatics. 2011 Jun 11;12:231. doi: 10.1186/1471-2105-12-231 (PMC3212839; doi:10.1186/1471-2105-12-231)
Supplement: Additional file 5 — Manual of our programs: simreseq and testassoc. Manual of our programs: simreseq and testassoc. [file 1471-2105-12-231-S5.PDF]

Supplementary Information: Software description

Su Yeon Kim

May 10, 2011

Contents

1 Introduction **1**
1.1 Platforms and Installation 1

2 Simulating SNPs in re-sequencing data: `simreseq` **2**
2.0.1 Introduction 2
2.0.2 Commands and options 2
2.0.3 Output files 3

3 Estimating MAFs and testing for association: `testassoc` **3**
3.0.4 Introduction 3
3.0.5 Commands and options 4
3.0.6 Output files 4

4 Run examples **6**
4.1 Generate simulation files 6
4.2 Testing for association 6

1 Introduction

This file is the manual for the programs `simreseq` and `testassoc`. These two seperate programs are in folder called `src` along with their "friends". This is a supplemental material for the article called "*Estimation of allele frequency and association mapping using next-generation sequencing data*".

Typing program name with any arguments, will give a list of options along with an example.

1.1 Platforms and Installation

These program are implemented in `c` and has been developed and tested on `POSIX UNIX` platforms, namely ubuntu 10.04 64bit using GNU tool chain 4.4.1, and Mac OS X 10.3 using GNU tool chain 4.1 and Mac OS X 10.6.6 using GNU too chain 4.5.0.

Under the directory containing the source codes, view `Makefile` and modify it if necessary. Type `make`. It will generate a program called `simreseq` and `testassoc`.

```
Compilation of programs
cd src/
make
```

For other compilers, eg `icpc,sun` these can be parsed to the compilation process as `CC`. Flags used for linking etc, are `CFLAGS`.

```
Compilation of programs (alternative compiler)
cd src/
make CC=icc #for Intel (R)
```

2 Simulating SNPs in re-sequencing data: `simreseq`

2.0.1 Introduction

This program simulates SNPs in re-sequenced regions. At each SNP, for each sequenced individual, it outputs the counts of four types of read bases (A C G T). Simulation can be done for two settings. One is for inferences in population genetics in which the minor allele frequency (MAF) at each site is drawn from a standard neutral coalescent model with a population size of 10,000. Another is for association studies in which MAFs of cases and controls are assigned using a multiplicative disease model. The prevalence of the disease, the combined MAF and relative risk need to be specified. Note that SNPs can be simulated under the null hypothesis (MAFs for cases and controls are the same) by setting relative risk at 1.0. For more details of description, see Methods in the main text.

2.0.2 Commands and options

Type `./simreseq`. It will describe input variables and file name specifications (see below). Each specification needs to be followed by an input file name or a specifying value. For example, `-efile` needs to be followed by the name of the file containing 4 by 4 sequence error rate matrix, and `-nloc` needs to be followed by the number of SNPs to simulate. The order of four nucleotides is A C G T in this program.

```
Commandline help
./simreseq
  ------> Simulate SNPs in re-sequencing data: version 1.00
  -> Input command:        ./simreseq
  -> ---------------------------------------------------
  -> (required infile) -efile: error rate matrix
  -> (required variable) -nloc: number of sites
  -> (required variable) -ncases: number of cases
  -> (required variable) -ncontrols: number of controls
  ----> note: for simulation under a standard neutral coalescent model,\
            only ncases+ncontrols matters
  -> (required variable) -dpcases: indivivdual sequencing depth for cases
  -> (required variable) -dpcontrols: indivivdual sequencing depth for controls
```

```
-> (required settings) -outdir: output directory
-> (required settings) -outputname: common name used for output files
-> (required variable) -iscasecontrol: indicator if sites are simulated\
         for association studies
----> note: if 'iscasecontrol' is 0, then sites are simulated under\
         a neutral coalescent model
---->      if 'iscasecontrol' is 1, then sites are simulated for association studies\
         in which cases and controls may have different MAFs
---->      in this case, minor allele frequency, relative risk, and\
          prevalence should be given
-> (optional variable) -maf0: minor allele frequency for disease SNP
-> (optional variable) -rr: relative risk for disease SNP
-> (optional variable) -prevalence: prevalence of the disease of interest

-> (example using a neutral coalescent model):
----> simreseq -efile errorfile -nloc 20 -ncases 5 -ncontrols 5 -dpcases 16\
 -dpcontrols 32 -outdir ./ -outputname Neutral -iscasecontrol 0

-> (example for case control study):
----> simreseq -efile errorfile -nloc 20 -ncases 5 -ncontrols 5 -dpcases 16\
          -dpcontrols 32 -outdir ./ -outputname Assoc -iscasecontrol 1 -maf0 0.2\
  -rr 2.0 -prevalence 0.1
 -> --------------------------------------------------
```

2.0.3 Output files

The parameter given to `-outputname` will serve as a suffix for the output files, these will be prefixed with `data` and `geno`

`geno` The file prefixed with `geno` contains simulated genotypes as a matrix. Each row corresponds to each SNP and each column corresponds to each individual. Three genotypes are denoted by the number of minor alleles, i.e., '2' implies homozygote of minor alleles.

`data` The file prefixed with `data` contains major and minor allele and counts of four nucleotides (orderd as A C G T). Each row corresponds to each SNP. At each SNP, the first two columns specify the major and minor alleles. For practical purposes, these alleles are shown as integer values: 0 (A), 1 (C), 2(G), and 3(T). The following columns are the simulated counts of four types of read bases. Each individual's data occupies four consecutive columns.

For both outputs, data from controls correspond to the first half of the columns (except major and minor allele columns) and data from cases correspond to the last half columns.

3 Estimating MAFs and testing for association: `testassoc`

3.0.4 Introduction

This program first computes genotype likelihoods using the counts of four nucleotides that are obtained by summarizing individual sequencing data at each

site (for details, see Methods in the main text). Then using the genotype likelihoods, it estimates MAFs and computes association test statistics based on genotype calling methods and maximum likelihood (ML) methods. For details of estimation of MAFs and test for association, see Results in the main text.

3.0.5 Commands and options

Type `./testassoc`. It will describe input variables and filename specification (see below). Each specification needs to be followed by an input file name or specifying value. For example, `-dfile` needs to be followed by the file name containing data matrix of counts of four nucleotides, and `-nloc` needs to be followed by the number of SNPs in the input data matrix. Note that in the data matrix, program assumes the first half columns are from controls and the last half are from cases.

The `readformat` option specifies whether the data matrix includes major and minor columns. This option is only for practical purposes and does not affect analyses results. In the simulated data, the true major and minor alleles are known and contained in the data matrix. To read this file, set `readfomat` as 0. From real data, only counts of four nucleotides would exist and therefore the data matrix does not contain major and minor allele columns. Set `readformat` as 1 to read this data.

Compilation of programs

```
./testassoc

 ------> Testing for association: version 3.400
 -> Command:        ./testassoc

 -> (required infiles) -efile: error rate matrix
 -> (required infiles) -dfile: data file
 -> (required variables) -ncases: number of cases
 -> (required variables) -ncontrols: number of controls
 -> (required variables) -nloc: number of sites
 -> (required settings) -outdir: output directory
 -> (required settings) -outputname: common name used for output files
 -> (optional variables) -readformat: format of the read-base counts data
 -----> readformat 0: data matrix contains two columns indicating major and minor alleles
 -----> readformat 1: data matrix contains only counts of four bases (A C G T)

 -> (example testing snps generated under the null):
 -----> testassoc -efile errorfile -dfile dataNull -ncases 5 -ncontrols 5 -nloc 20\
          -outdir ./ -outputname outNull -readformat 0
 -> (example testing snps generated under an alternative):
 -----> testassoc -efile errorfile -dfile dataAlt -ncases 5 -ncontrols 5 -nloc 20\
          -outdir ./ -outputname outAlt -readformat 0
 -> --------------------------------------------------
```

3.0.6 Output files

In total, nine output files are to be produced. For example, with prefix of `outAlt`, the list of output files are as follows. The last file is generated only for computational purpose, so can be ignored.

```
outAlt_geno_fl0.0
outAlt_geno_fl1.0
outAlt_geno_fl2.0
outAlt_tabgeno_fl0.0
```

```
outAlt_tabgeno_fl1.0
outAlt_tabgeno_fl2.0
outAlt_lrt_obs
outAlt_lrt_unkwn
outAlt_pats
outAlt_obsmaps
```

For genotype calling methods, outputs exist for three thresholds: 0.0, 1.0 and 2.0. With a specified threshold f, the genotype is called if

$$\log_{10}\left(\frac{\text{largest genotype likelihood}}{\text{second largest genotype likelihood}}\right) > f.$$

Otherwise, genotype is treated as missing and marked as '-2' in the output files. Using the called genotypes, MAFs are estimated, and G-statistic and Armitage trend test statistic are computed. Specifically, at each of the three thresholds, two output files exist, one containing called genotypes (eg. outAlt_geno_fl1.0) and the other containing the estimated MAFs, G-statistics (eg. outAlt_tabgeno_fl1.0). The latter file has 13 columns:

1. MAF under the null
2. MAF of cases
3. MAF of controls

4-11. Summary of called genotype counts

12. G-statistic
13. Armitage trend test statistic

For maximum likelihood methods, two output files exist, one for assuming known minor allele (outAlt_lrt_obs) and the other without assuming a specific minor allele (outAlt_lrt_unkwn). In the former case, we take the second most common neucleotide (across all individuals) at each SNP as the known minor allele for that SNP. Each output file contains six columns:

1. MAF under the null
2. MAF of cases
3. MAF of controls
4. Log-likelihood under the null hypothesis
5. Log-likelihood under the alternative hypothesis
6. LRT statistic

4 Run examples

4.1 Generate simulation files

First we validate our errorfile

Validate our error transition matrix

```
$cat errorfile
0.0 0.005 0.0025 0.0025
0.005 0.0 0.0025 0.0025
0.0025 0.0025 0.0 0.005
0.0025 0.0025 0.005 0.0
```

This looks fine so let's generate data under the NULL-hypothesis

Calculate "counts" files

```
$./simreseq -efile errorfile -nloc 20 -ncases 5 -ncontrols 5\
 -dpcases 16 -dpcontrols 32 -outdir ./ -outputname Null\
 -iscasecontrol 1 -maf0 0.2 -rr 1.0 -prevalence 0.1
```

4.2 Testing for association

Let's test the data generated under the null.

Running testassoc

```
./testassoc -efile errorfile -dfile dataNull -ncases 5\
 -ncontrols 5 -nloc 20 -outdir ./ -outputname outNull\
 -readformat 0
```
